# Exploring EEG Characteristics to Identify Emotional Reactions under Videogame Scenarios

**DOI:** 10.3390/brainsci11030378

**Published:** 2021-03-16

**Authors:** Laura Alejandra Martínez-Tejada, Alex Puertas-González, Natsue Yoshimura, Yasuharu Koike

**Affiliations:** 1 FIRST Institute of Innovative Research, Tokyo Institute of Technology, Yokohama, Kanagawa 226-8503, Japan; yoshimura@pi.titech.ac.jp (N.Y.); koike@pi.titech.ac.jp (Y.K.); 2 System Engineering and Computation School, Universidad Pedagógica y Tecnológica de Colombia, Santiago de Tunja 150007, Colombia; alex.puertas@uptc.edu.co; 3 Department of Advanced Neuroimaging, Integrative Brain Imaging Center, National Center of Neurology and Psychiatry, Kodaira, Tokyo 187-8551, Japan; 4 PRESTO, JST, Kawaguchi, Saitama 332-0012, Japan; 5 Neural Information Analysis Laboratories, ATR, Kyoto 619-0288, Japan

**Keywords:** emotion recognition, classification, correlation, human-computer interaction, EEG signals, regression, videogame

## Abstract

In this article we present the study of electroencephalography (EEG) traits for emotion recognition process using a videogame as a stimuli tool, and considering two different kind of information related to emotions: arousal–valence self-assesses answers from participants, and game events that represented positive and negative emotional experiences under the videogame context. We performed a statistical analysis using Spearman’s correlation between the EEG traits and the emotional information. We found that EEG traits had strong correlation with arousal and valence scores; also, common EEG traits with strong correlations, belonged to the theta band of the central channels. Then, we implemented a regression algorithm with feature selection to predict arousal and valence scores using EEG traits. We achieved better result for arousal regression, than for valence regression. EEG traits selected for arousal and valence regression belonged to time domain (standard deviation, complexity, mobility, kurtosis, skewness), and frequency domain (power spectral density—PDS, and differential entropy—DE from theta, alpha, beta, gamma, and all EEG frequency spectrum). Addressing game events, we found that EEG traits related with the theta, alpha and beta band had strong correlations. In addition, distinctive event-related potentials where identified in the presence of both types of game events. Finally, we implemented a classification algorithm to discriminate between positive and negative events using EEG traits to identify emotional information. We obtained good classification performance using only two traits related with frequency domain on the theta band and on the full EEG spectrum.

## 1. Introduction

Emotion recognition’s studies using electroencephalography (EEG) signals have been increasing due to the potential of building brain computer interphases (BCIs) systems for assistive and companion computing solutions [1,2]. The ability to dynamically detect users’ emotional states is crucial to design adaptive emotion recognition system, this is why researchers use physiological signals to evaluate the emotional changes through time (as EEG, electromyography, galvanic skin responses, eye movement, respiration, body movement, etc.). Some of the works focus only on the study EEG under human computer interaction (HCI) scenarios, due to the potential of including cognitive information in the biofeedback loop to build more intuitive systems. One potential field of study are videogames (an example of HCI scenarios), defined as digital interactive tools with the ability of elicit emotions in players, using resources as storytelling, game mechanics, aesthetics and digital media (as music, sounds, pictures, videos). Videogame play is cognitively demanding, and emotionally arousing and engaging, eliciting both positive and negative emotions on the players [3]. With this potential, some emotion recognition works have used videogames as an emotional stimulus, mainly under game development scenarios to target specific emotions as boredom, stress, fear or engagement [4,5,6,7,8]. The study of EEG signal on gaming scenarios can give an approximation on how emotions manifest on an HCI scenario level, not only for game related events, but also for cognition, training, and BCI applications [9,10,11,12,13]. For this study, we wanted to identify works where videogames are used as an emotional stimulus in emotion recognition experiments, along with EEG signals to identify and assess participants’ emotional reaction. In this section, we described some of the works developed in the field, and in Table 1, we summarized the characteristics of the different works.

In [14], a dataset using EEG and videogames for emotion recognition studies was build, using 4 different commercial videogames that represented four emotional states (Train Sim World: boring, Unravel: calm, Slender—The Arriva: horror, Goat Simulator: funny). A total of 28 participants played the four videogames and signals from 16 electrodes were recorded (using the 14 channel EMOTIV EPOC+ MobileEEG device (EMOTIV, San Francisco, CA, USA)). The participants rated the games after playing using arousal and valence scores. To classify emotions, time-frequency traits were extracted, and used features for machine learning algorithms to perform multi-class and binary classifications. They calculated detrended fluctuation analysis, Hjorth features, average energy of wavelet coefficients, Shannon entropy, logarithmic energy entropy, sample entropy, multiscale entropy, standard deviation, variance and zero-crossings from beta and gamma bands, and obtained the best accuracy classification from channels AF3, F4 for multiclass classifications (80% and 82% respectively). For binary classification—AF3, AF4 (86% and 87%).

In [15], EEG signals were recorded to perform emotion recognition using a virtual reality environment emulating architectural rooms displayed by 360° panoramas during time windows of 90 s. They recorded electrical activity from frontal (Fz, F3 and F4), central (Cz, C3 and C4) and parietal (POz, P3, and P4) electrodes. Four base-scenarios were designed modifying different parameters of three design variables: illumination, color, and geometry, to achieve four different emotional states. They calculated frequency traits from θ (4–8 Hz), α (8–12 Hz), β (13–25 Hz), γ (25–40 Hz) bands, and functional connectivity traits. The most important features selected by their recursive feature elimination-support vector machine model derived from the EEG functional connectivity analysis, suggesting that cortical functional connectivity provides effective correlations of emotions.

In [16], two android games were selected as emotional stimuli: Candy Crush Saga as a leisure game, and Stickman Archers as a violent game, to elicit 6 different emotions: happiness, sadness, surprise, disgust, anger and neutral state. They recorded the EEG signals using a 21-channel Nihon Kohden EEG system. The experiment was carried out over 30 days for each subject, and on each day, five experimental trials were conducted, where each participant played a given android game for 10 min duration. Using facial images captured during game playing, they identify the six different emotional targets during each gameplay and then analyzed the EEG signals to identify the activated brain regions that exhibited emotion activation. They observed that pre-frontal, frontal and temporal brain region remains highly active during the five emotions’ arousal state (except neutral), whereas, only occipital lobe activation was found during neutral (no cognitive task/neutral) condition.

In [17], propose an adapting method for Tetris game difficulty according to player’s emotions assessed from physiological signals, including 19 channels from EEG. The participants performed different session where they played the game in a 5 min time-window. In their analysis, each game difficulty was rate with an emotional reaction using questionnaires. They found several features in the theta and beta bands that were significantly different among three difficult conditions (easy, normal, hard) related to electrodes in the left (theta: C3, T7, P3, P7, O1, beta: Fp1, P7, O1), middle (theta: Fz, Cz, beta: Cz), and right (theta: F4, C4, T8, O2, beta: C4, T8, P8, O2) areas of the brain.

In summary, previous works used EEG signals and different videogames or virtual environments to elicit emotions and perform emotion recognition, focusing on a continuous approach using arousal/valence scores, or on a discrete approach using few categorical emotions to label affective states. However, one of the main common limitations that the works present are the time windows for emotional reaction analysis, because emotions are dynamic expression that manifest as a reaction according to a specific stimulus, long time windows prevent the analysis of the emotional reaction transitions when there are different task or events inside the videogame. In addition, using only self-assessment responses to evaluate the emotional experience does not allow to study specific reactions that occur inside the gameplay time window, these reactions are important if we want to identify the dynamics of the EEG signals when an emotional reaction occurs. Our hypothesis is that it is useful to consider game time events, related to tasks or game mechanics, to analyze and understand how the participants reacts together with the self-assessment to evaluate of the emotional experience.

We wanted to explore the characteristics of the EEG signal and the correlation between the emotional states reported by the participants through self-assessment responses and the game events intended to elicit positive and negative emotions. While including the analysis of EEG traits and game time events, together with the self-assessment responses, on two different time scenarios, we wanted to identify:

- The EEG traits that correlated with the emotional self-assessment responses for all the participants, in an individual approach after playing a videogame level.
- The performance of machine learning regression methods to predict emotional self-assessment responses.
- The relation between the number of game events from different game levels and the arousal/valence responses from the participants.
- The characteristics of the EEG signal in the presence of game time events related with emotional reactions.
- The possibility to classify those game events using only EEG traits to assess emotional reactions inside a game play time window.

The article’s structure is as follows: in the methodology section we described the videogame used as an emotional tool, the participants’ sample, the type of information gathered (self-assessment emotional answers and game events), the EEG signal preprocessing, and EEG traits calculation. In the result section, we analyzed and discussed the correlation between arousal and valence scores with EEG traits, and implemented a regression algorithm to predict these arousal–valence values from EEG information. In addition, we analyzed and discussed the correlation between game events and the calculated EEG traits as another approach to identify reactions to emotional content under HCI scenarios, and implemented a classification algorithm to identify positive and negative events from EEG traits. Finally, we discussed the results and the limitations of our study.

## 2. Materials and Methods

### 2.1. Emotional Stimuli Tool

We developed a 2D platform game, framed in a space theme: the participant controls a spaceship to perform different task like rescue astronauts, collecting coins, and avoiding asteroids or aliens during different game levels, with the main goal of achieve the best possible score in each of the game levels (details are described in Appendix A). The spaceship is located in the left part of the screen (Figure A2) and, the movement is limited to the *y* axis only with 3 controls: the first one for moving the spaceship to the upper part of the screen with the up arrow key, the second one for moving the spaceship to the lower part of the screen with the down arrow key, and the third one to increase the tokens’ scroll speed with the right arrow key, all of these functions are controlled with one hand.

**Figure A1:**
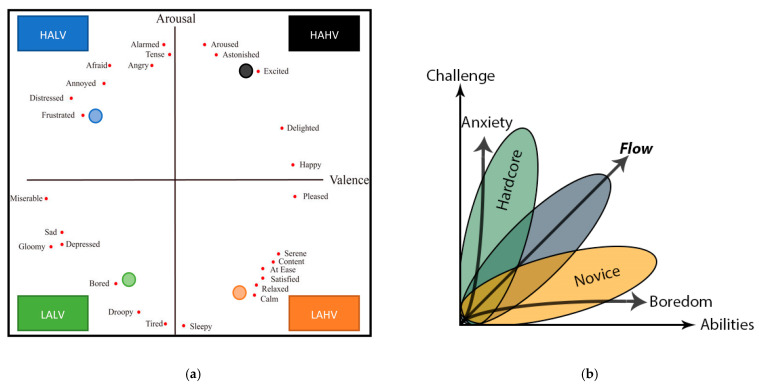
(**a**) Russell’s circumflex model of emotions, where the emotions selected are highlighted in the different quadrants of the two-dimensional plane [18]. (**b**) Player in-game flow experience [57].

**Figure A2:**
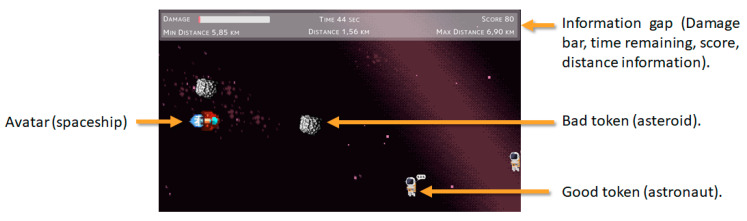
Game interface with the player’s avatar and different tokens deployed. The information gap is located in the upper part of the screen containing information about the spaceship’s damage, time remaining, the score, the distance traveled and an upper and a lower distance threshold (game mechanics).

We designed three stages: Stage 1 (Elysian) is the basic stage that will include the elements described before (astronauts, asteroids and coins). For Stage 2 (Asgard V) and Stage 3 (Eden), we changed the aesthetics of the background environment and added a distinctive new game element, represented as a new negative token. Each stage contains 8 levels that target 4 different emotional states according to Russell’s circumflex model of emotion [18] (2 levels per each dimensional emotion that we are trying to elicit, Figure A1a). The emotional states are: frustrated (high arousal and low valence—HALV) elicit by: H—hard, OA—only asteroids; excited (high arousal and high valence—HAHV) elicit by: N—normal, SU—speed up; calm (low arousal and high valence—LAHV) elicit by: E—easy, WS—without speed; and bored (low arousal and low valence—LALV) elicit by: SD—speed down, WT—without tokens. Each game level has its own music tracks, and each token has a distinctive sound to enhance the targeted emotion according to each level [19,20,21,22]. Characteristics of each game level are shown in Table A1.

**Figure 1:**
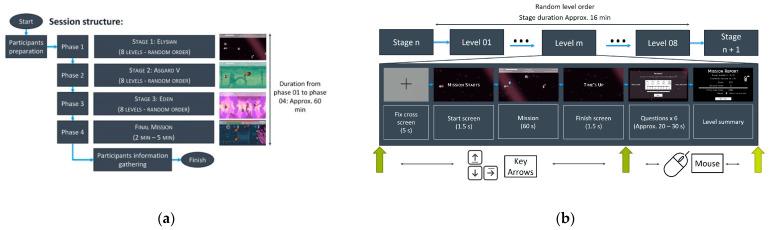
Game structure for one experiment session and level structure: (**a**) game structure composed by 3 phases containing the 3 designed stages; (**b**) game level’s structure.

**Figure A3:**
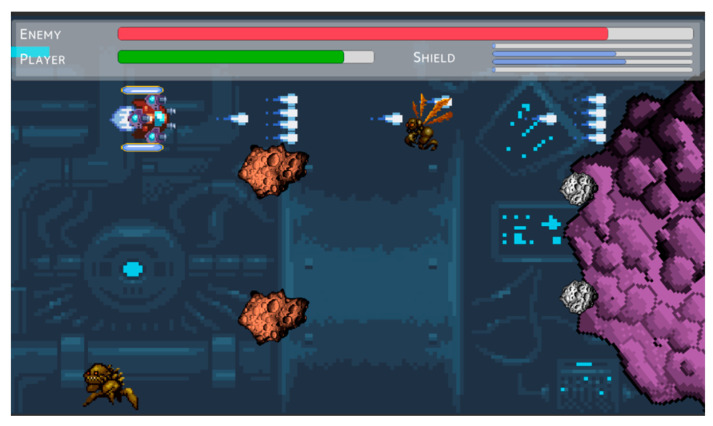
Game interface for the final mission with the spaceship and different tokens deployed.

The game is divided in 4 phases (Figure 1a), for the first, second and third phase, we presented to the participant the Stage 1 (Elysian), Stage 2 (Asgard V) and the Stage 3 (Eden), respectively. For the fourth and final phase, we presented to the participant a final mission (Figure A3). In Stages 1, 2, and 3, eight game levels are presented in a random order, and each game level, is composed of: a cross fixation screen displayed for 5 s, the game level in which the participant interacts directly with the virtual environment for 60 s, the questionnaires to evaluate the emotional and game experience according to each game level, and a game score feedback screen where a brief summary of the level performance (Figure 1b). With this structure, the participants will evaluate their emotional experience immediately after they stop playing each level, and will allow them to assess their performance without worrying about the emotional report. Each stage takes approximately 16 min to be completed, and the whole game takes about 1 h.

### 2.2. Type of Acquire Information

#### 2.2.1. Emotional Questionnaires

To record the emotional experience from the participants after each game level, we designed 2 videogame screens with emotional questionnaires. We used two variables from the self-assessment manikins (SAM) [23] (scales ranging from 1 to 9): arousal (1 being “very calm” and 9 being “very excited”), and valence (1 being “very negative” and 9 being “very positive”). These questionnaires are manipulated using a mouse, virtual sliders, and buttons, the scales are represented with a guiding bar, numbers, and a graphic guide. The answers gave by the participants corresponded to a game-level assessment in a 60 s time-window.

#### 2.2.2. Game Events

Inside each game level, the participant will interact with 2 kind of events: positive and negative. We defined and gathered time information of the game events: first positive events, which happened when the participant’s avatar collided with a token that adds points to the overall score on the game level (is an event which aims to be pleasant for the participant), examples of positive tokens are: astronauts (as an emphatic element), coins, and power ups (as an identifiable element of reward). Second, negative events, which happened when the participant’s avatar collided with a token that takes points from the overall score on the game level (is an event which aims to be unpleasant for the participant), examples of negative tokens are: asteroids and enemies (as elements of danger and damage). Inside each game we recorded the time when the event happened and the type of event, and after each game level, we recorded the total amount of positive and negative events. Each event is also presented with an audio cue related to a sound produced when the participant collides with a token.

#### 2.2.3. Electroencephalography

EEG signals have proven to achieve higher classification accuracy for emotion recognition under laboratory conditions [24], and have come along as a useful tool that describes how cognition and emotional behavior is related in a physiological level. The limbic system is known for controlling basic motivations, including emotions, also, affect-related processing in the human brain is distributed across the brainstem, limbic, paralimbic, and neocortical regions [25].

##### Signal Preprocessing

The EEG signals were acquired from 66 channels (64 EEG channels and 2 earlobe references) using a Biosemi Active Two amplifier system with active sensors (Biosemi, Amsterdam, Netherlands), at a sampling rate of 2048 Hz. The EEG electrodes were positioned on the head according to the International 10–20 system. ActiView was used to monitor and setup the signals prior recording, and the LSL’s Biosemi application software was used to record the signals.

Lab stream layer (LSL, Swartz Center for Computational Neuroscience, University of California, San Diego, CA, USA) recorded the data’s streams that contained the actual sample data related to the signal values, and event markers that came from the videogame, together with the timestamp for each sample that is read from a local high-resolution clock of the computer. To extract the signal related to each level, we identified the start level and finish level markers events of each level, generated from the game environment, and extracted the signals portion related to these time windows.

Data were processed using MATLAB R2019b (The MathWorks, Inc., Natick, MA, USA), we reference the 64 channels’ signals to the ears and applied a finite impulse response (FIR) notch filter at 50 Hz, then we applied a FIR high pass filter at 1 Hz and a FIR low pass filter at 40 Hz, then down-sampled the signal at from 512 HZ to reduce the computational cost. Then we inspected and reject the noisy channels and referenced the signals to the average of the channels. Finally, we applied independent component analysis (ICA) [26,27,28], and inspected each of the components to manually reject the ones related with noise and artifacts (eye movement, blinks and muscular activity) [2,29,30].

##### Signal Traits

Traits are characteristics of the signal that describe the behavior according to different analysis domains, on this case, we considered 2 types of traits that have proved to be related with emotions [2]. The list of the extracted features is summarized in Table 2, the formulas and process to calculate each of the traits can be found in each of the works cited in the table.

- Statistical (time domain) features: are statistical parameters of the physiological signal time series, over a relatively a long-time window.
- Frequency domain features: considers a frequency spectrum and different frequency bands related to signal activation produce by a specific stimulus.

We calculated 8 time-domain features (mean, standard deviations, ratio max/min, skewness, kurtosis, activity, mobility, complexity) and 10 frequency-domain features (power spectral density—PDS, differential entropy—DE for 5 frequency bands: theta, alpha, beta, gamma bands, and full frequency spectrum) for each EEG channel (18 × 64 = 1152); in addition, we calculated 15 frequency-domain features (Power spectral asymmetry—PS-ASM, differential asymmetry—DASM, rational asymmetry—RASM) for each of the 27 pair of electrodes (15 × 27 = 405). In total, we calculated 1557 EEG traits to be used for correlation analysis and predictions with emotional labels. For arousal and valence scores we considered a 60 s time window (related to the full gameplay of each of the 25 game level), and for game time-events we considered a 500 ms time window (from the occurrence of all game events in each of the 24 levels).

#### 2.2.4. Participants

12 participants (female 4, male 8) with ages ranging from 25 to 43 (mean 32.10 ± 5.40) from the following countries: Japan, Macedonia, Greece, Canada, Filipinas, and Vietnam, participated in the study. The experiment was conducted in an individual scheme, at the Laboratory for Future Interdisciplinary Research of Science and Technology (FIRST) from Tokyo Institute of Technology. The experiment was approved by the ethics committee of the Tokyo Institute of Technology (Approval No. A20039) and conducted in accordance with the Declaration of Helsinki. The procedures were explained to each participant prior the experiment, and they were allowed to rehearse using 3 practice levels corresponding to the normal level from the first stage of the stimulus. The experiment took about 2 h and a half to be completed for each participant, 1 h and a half for the experimental setting and 1 h for playing the videogame. Participants were positioned sitting in a reclining chair in a sound-attenuated chamber, and instructed to look at the monitor positioned approximately 70 cm away from their eyes during the experiment. The videogame was presented to the participants using a 24” monitor, and the participants manipulated the videogame using a keyboard and a mouse. The chamber light was kept on during the experiment and participants were allowed to take breaks of unlimited time between each game level. To record the data, we used one computer with two sets of screens, keyboards and mice: the first set was used to show the stimulus and allow the participant to control the experiment task. The second set was used to monitor the signal acquisition and the participant’s performance during the task. The participants’ signals were recorded simultaneously with different software, and synchronized with LabRecorder-1.12 (Lab Stream Layer (LSL) Swartz Center for Computational Neuroscience, University of California, San Diego, CA, USA) which gathered the EEG signals and the trigger events from the videogame. After signal inspections, information from 2 participants were rejected due to the high artifact contamination.

## 3. Results

### 3.1. Self-Assessment Responses and Game Events

#### 3.1.1. Arousal–Valence Dispersion

**Figure 2:**
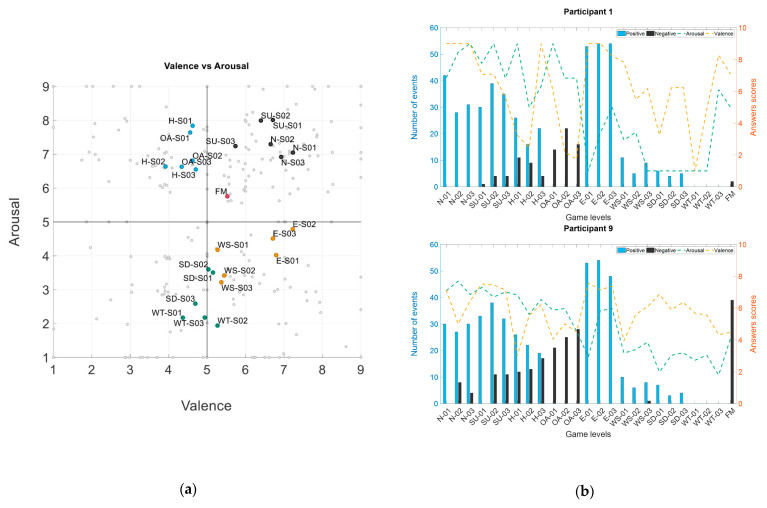
Participants’ information for self-assessment and game events. (**a**) Arousal–valence dispersion. Levels names: N—normal, S01—Stage 01, S02—Stage 02, S03—Stage 03. The colors are related to the emotional quadrants that we intent to induce: high arousal and high valence (HAHV)—black, high arousal and low valence (HALV)—blue, low arousal and high valence (LAHV)—orange, low arousal and low valence (LALV)—green. (**b**) Total amount of positive and negative events obtained by each participant across all the game levels along the arousal and valence responses.

In Figure 2a, the arousal and valence scores from all the participants are represented in a two-dimensional plane. The participants reported different emotional states within each game level, were the obtained means are related to a generalized emotional experience, they work as a reference on how each game level elicited the emotional states. In Table 3, we summarized the mean and standard deviation values for each of the game levels.

For normal and speed up levels (N, SU, black dots), HAHV scores were reported throughout the three stages. It is possible to see that the mean values are similar, and the majority of the distribution is located in the HAHV quadrant, leading to infer that the excited emotion was achieved over the six levels. For hard and only asteroids levels (H, OA, blue dots), the arousal scores aimed to represent HALV responses, leading to infer that the frustrated emotion was achieved over the six levels.

For easy and without speed levels (E, WS, orange dots), LAHV responses were reported by the participants. For easy levels, HV scores were higher than the without speed levels’ valence scores. Without speed levels’ mean reference values are located close to the neutral valence value. In general, the easy levels’ mean reference values showed that calm emotion was induced by the six levels, and the without speed levels’ mean reference values showed that bored and calm emotion were induced in the participants. For speed down and the without tokens levels, LA responses were reported by the participants. Valence responses distribution is located between the two valence quadrants making the mean reference values to be located in the LV quadrant but close to the neutral valence value. In general, bored emotion was achieved over the six levels. Finally, for the final mission level, most of the answer distribution was located in the HAHV quadrant, leading to infer that excitement is the emotion felt by the participants while playing this last level.

#### 3.1.2. Game Events

We inspected the number of positive and negative events obtained by each of the participants. In Figure 2b, we show an example of different participant’s performance according to the number of positive and negative events in each level, along the arousal and valence responses reported. Participant 1 had a lower number of negative events across the game levels, in contrast, Participant 9 had a higher number of negative events in levels aimed to induce HALV and in the final game level. It is possible to see that the number of positive events is higher than the number of negative events, this is understandable because of the objectives proposed by the virtual environment of collecting positive tokens and avoid collision with negative tokens, however, there is still a high number of negative events that allowed the study of emotional reaction.

### 3.2. Analysis from Self-Assessment Responses and EEG Traits

#### 3.2.1. Spearman’s Correlation of EEG Traits with Arousal and Valence Scores

To study the relation between the self-assessment responses and the EEG traits, we performed Spearman’s correlations and reported the EEG traits with a strong (equal or higher than |0.5|) correlation’s score and significant p-value (*p* < 0.005). We performed Spearman’s correlation between the 1557 EEG traits and the arousal/valence scores given by the participants after completed each of the game levels (60 s time window).

On Table 4a, we show the number of traits correlated for each participant. We found that the lower amount of EEG traits for arousal was 106 (6.8% of the total number of calculated traits) and the higher amount was 287 (18.43%). For valence, the lower amount of EEG traits was 7 (0.45%) and the higher amount was 323 (20.74%). On average, the number of traits correlated with arousal was higher than the number of traits correlated with valence (only for Participant 5 the number of correlated traits for valence was higher than for arousal).

In contrast, when we considered common correlated EEG traits, few common traits had a correlation for the participants’ majority and arousal answers, it is possible to see from Table 4b, that when we consider more participants the number of common traits decrease. From the 1557 EEG traits calculated, only 3 were common correlated for all the 10 participants, and 461 EEG traits were unique among the 10 participants (461 traits had only one occurrence when the correlated EEG traits of the 10 participants were inspected). For valence answers, fewer common traits were found (the higher number was 4 participants with 1 common trait), and 480 traits had only one occurrence when the correlated EEG traits of the 10 participants were inspected.

**Figure 3:**
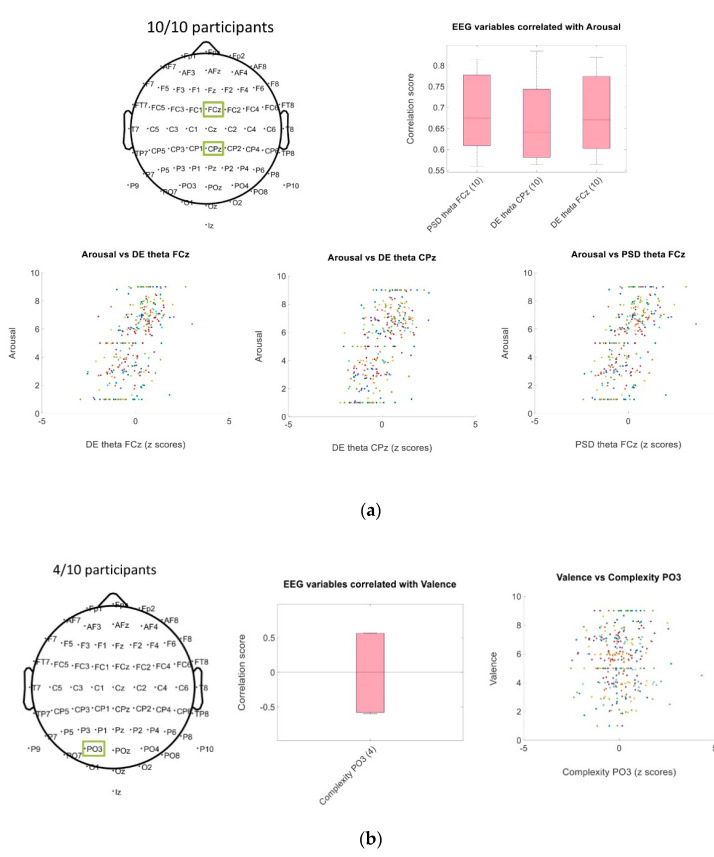
Spearman correlation score’s dispersion of traits common for the participants. (**a**) Traits correlated with arousal scores. The reported traits have positive rho scores with a mean value above 0.6. (**b**) Trait correlated with valence scores, for 4 participants only one trait had a strong correlation, in this case, the correlations had positive and negative rho scores among participant whit scores no higher than 0.6.

We explored the correlation scores of the common traits among participants. We identified that PSD and DE traits on the theta band for channels in the frontal (F), central (C), and parietal (P) regions: FCz, CPz, Cz, FC1, FC2, C1, CP1, CP2 had positive correlation for the majority of the participants. In Figure 3a, we showed the traits correlated with arousal for all the participants and the dispersion of each trait. In contrast, in Figure 3b, the only common correlated trait with valence for 4 participants is shown (complexity of PO3 channel had both positive and negative correlations and lower rho scores compare with the traits correlated with arousal), from the dispersion is possible to identify that is more difficult to identify a clear pattern. We showed that there is a higher number of traits that correlated with arousal scores than valence scores across all participants. Arousal scores are related with traits in the theta frequency band and electrodes located in the frontal central, central, and central parietal regions.

We can infer that the trait correlations with arousal scores vary between participants due the individual difference on behavior and reaction to the emotional content, and how each individual approached the experimental task. In addition, as can be related with the time window to extract the different traits, during to each level, different physiological activations can occur depending on the events and challenges that each game level presents. However, despite the difference between participants, it is possible to see that arousal scores can be describe by different EEG signal traits. There are other traits that correlated in an individual approach for both arousal and valence scores, for this reason, we decided to implement a regression algorithm to predict these scores and evaluate the performance on an individual level.

#### 3.2.2. Arousal and Valence Prediction Using Bayesian Ridge Regression Model

We wanted to identify the prediction performance of arousal and valence scores using the EEG traits, for this, we implemented Bayesian ridge regression model to make predictions using 25 observations per participant and EEG traits selected form the 1557 calculated. Bayesian regression techniques can be used to include regularization parameters in the estimation procedure, this is done by introducing uninformative priors over the hyper parameters of the model. For Bayesian ridge regression The loss function is augmented in such a way that not only minimize the sum of squared residuals but also penalize the size of parameter estimates [42].

**Figure 4:**
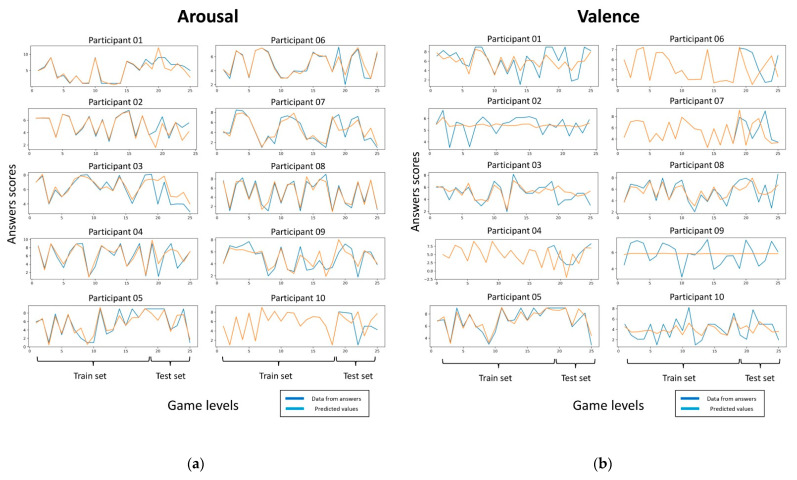
Performance of Bayesian ridge regression predictions for arousal and valence scores. (**a**) Arousal score values and predictions over the train and the test set. (**b**) Valence score values and predictions over the train and the test set.

We calculated the z scores of each trait to normalize the values, then, we split the dataset into train set (75%) and test set (25%), and performed feature selection for regression approaches, using mutual information [43] and grid search with repeated cross validation (splits = 10, repeats = 3, random state = 1) over the train set, with mean absolute error scoring. Traits selected as features for arousal and valence score are related with time domain (standard deviation, complexity, mobility, kurtosis, skewness), and PSD and DE from theta, alpha, beta, gamma, and all EEG frequency spectrum. Then, we trained the models over the train set using the features selected, with a repeated cross validation scheme (splits = 10, repeats = 3, random state = 1), and calculated the mean absolute errors (MAE) and the mean square errors (MSE) as performance scores. Finally, we tested the obtained model over the test set. We reported the results in Figure 4. For arousal, MAE had an average of 0.973 ± 0.316 on train set, and 1.199 ± 0.321 on test set, MSE had an average of 1.786 ± 1.122 on train set and on test set 3.186 ± 2.876, we achieved the best result for Participant 6 in the train set (MAE: 0.406 ± 0.299, MSE: 0.328 ± 0.406). For valence, the MAE had an average of 1.199 ± 0.321 on train set, and 1.670 ± 0.784 on test set, the MSE had an average of 2.504 ± 1.2112 on train set and on test set 4.680 ± 4.032, we achieved the best result for Participant 5 in the train set (MAE: 0.723 ± 0.317, MSE: 0.806 ± 0.655). From the prediction models’ implementation, we had better result for arousal scores than valence scores in both the train set and the test set (only Participant 5 got better results for valence than for arousal scores in both train and test set). In Figure 4, it is possible to see the regression’s prediction of arousal and valence values across all videogame levels (25 game levels or observations) for each of the participants. In general, is easier to find EEG traits that correlated and helps to describe the arousal answers given by the participants than valence answers.

### 3.3. Analysis from Time Related Events and EEG Traits

**Figure 5:**
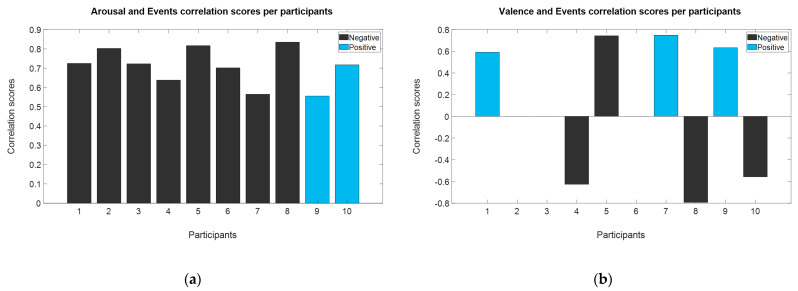
Correlation between arousal–valence scores and number of events per game level. (**a**) Arousal correlations. (**b**) Valence correlations.

We wanted to identify the relation between the game events and the arousal and valence answers. Although, we labeled the event as positive or negative according to the context of the videogame and the event that represented, the correlation allowed us to determine if those events have the same appraisal for each of the participants. To perform the correlation, we count the amount of both events (positive and negative) in each of the game levels per participants, then we used Spearman’s correlation to identify which even type correlated with the answers gave by the participants. On Figure 5, we showed: (a) the correlation score between arousal and number of events, and (b) the correlation score between valence and number of events. For arousal scores, the number of negative events had a positive correlation across the majority of the participants, only for Participants 9 and 10, positive events correlated positively. For valence score, strong correlations were found for negative events (Participants 4, 5, 8 and 10) and for positive events (Participants 1, 7, 9). Presence of negative events can induce higher levels of arousals responses; this can be due to the nature of each of the designed levels. With the results, we can infer that is possible to identify emotional reactions thought the identifications of game events.

#### 3.3.1. Spearman’s Correlation of EEG Traits with Game Events

To study the relation between the game time events and the EEG traits, we performed Spearman’s correlations and reported the EEG traits with a strong (equal or higher than |0.5|) correlation’s score and significant p-value (*p* < 0.005). First, we excluded the game events that were too close from another event (if an event had another event inside the 500ms time window, that event was excluded from the analysis). We identified that, for all 10 participants, the excluded events were less than 15% of the total positive events, and less than 30% of the total negative events. The final number of events used in our analysis is showed in Table 5.

**Figure 6:**
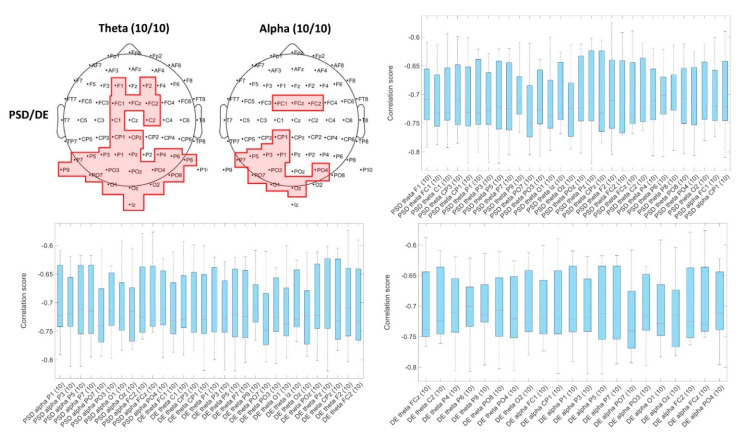
EEG traits correlated with game events. Theta band’s PSD and DE from electrodes on the occipital and central brain region and, alpha band’s PSD and DE from electrodes on the frontal-central and the occipital brain regions. The EEG traits had a negative correlation with positive events and positive correlation with negative events.

The Spearman’s correlation was performed between the 1557 EEG traits and the game events inside each of the game levels. We calculated the EEG traits from Table 2, on a signal portion extracted from a time window of 500 ms (0 s at event onset). We found that there is a high number of common traits for all the participants. PSD and DE traits of theta and alpha bands for all the EEG channels except of P2 correlated negative with positive events and positively with negative events. With lower rho scores, PSD traits of beta band for all EEG channels except of: Fp1, AF7, F7, F5, FT7, FC5, Fpz, AF8, F8, and P2; and DE traits of beta band for all EEG channels except of: Fp1, AF7, F7, F5, FT7, FC5, Fpz, AF8, F8, P2, Fp2, AF8, correlated negative with positive events and positively with negative events. As, example in Figure 6, we showed the traits and the topographical plots of channels with a rho scores stronger than 0.7 (rho ≥ |0.7|), it is possible to identify that the PSD and DE traits related with the theta (channels: F1, F2, FC1, FC2, FCz, C1, C2, CP1, CPz, P1, P3, P4, P5, P6, P7, P8, P9, Pz, POz, PO3, PO4, PO7, PO8, O1, O2, Oz, Iz) and the alpha band (channels: FC1, FC2, FCz, CP1, P1, P3, P5, P7, PO3, PO7, PO4, O1, Oz) had a negative correlation with positive events (positive correlation with negative events).

**Figure 7:**
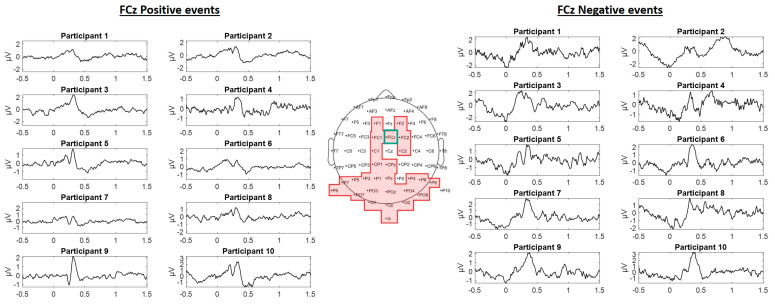
Time domain plots of FCz channel’s signals with a time window of 2.0 s, event onset at 0 s, as an example of patterns found on EEG signals in presence of game events for each of the participants.

We inspected the nature of the channels’ signal that correlated with the different events, we extracted the channels’ signals portion around the analysis time window (0.5 s from the event occurrence), and obtained the mean values of the channel’s amplitude. We identified some event related potentials (ERP) activation that manifested in the presence of an event for all the channels correlated with positive and negative events. In Figure 7, we showed as an example the FCz channel signal between a time window of 2.0 s (0.5 s before the event onset and 1.5 s after the event onset). For positive events, the signal exhibits similar characteristics of an ERP P200 (200 milliseconds and positive amplitude), for negative events, the signal exhibits an ERP activation immediately after the presence of the event.

The presence of ERP components, are evidence of participants reacting of the occurrence of game events, the frequency characteristics showed a distinction between the nature of the events that were distinctive according to each participant. With this information we wanted to implement a classification algorithm to distinguish between positive and negative events using the correlated EEG traits.

#### 3.3.2. Game events Classification using Ensembling Methods

The ratio between the total amount of positive and negative events acquired during the full gameplay is not even, the number of positive events was higher than the number of negative events (Figure 2b, and Table 5), because of this, we decided to implement a classification model for imbalance data on the classes. In ensemble classifiers, bagging methods build several estimators on different randomly selected subset of data. Ensemble methods use multiple learning algorithms to obtain better performance, bagging methods work by building multiple estimators on a different randomly selected subset of data, allowing to train the classifier that will handle the imbalance without to under-sample or oversample manually before training [44].

First, we randomly delete some of the observations from the majority class (undersample the positive events), to obtain the same number of observations from the minority class (negative events). Then, we performed feature selection with the under-sampled dataset using recursive feature elimination with cross validation and a support vector classification algorithm (linear kernel, regularization C = 100). Then, we split the dataset into Train set (75%) and Test set (25%) with the selected features. Then, we implemented a balanced bagging classifier using decision trees as estimator, with 10-fold cross validation and the selected traits on the train set (without under-sampling dataset).

In the Table 6, we showed the classifier’s performance with accuracy (Acc), F1, and area under the curve (AUC) scores. From the scores it was clear the good performance of the classifiers to discriminate between positive and negative events, the features selected from the majority of the participants were DE from the theta band and form the full EEG frequency spectrum, only for Participant 1 the traits selected were: DE theta (F1, C1, PO3, CPz, AFz, Fz, F2, FCz, CP2), DE alpha (F1, CPz, F2), DE from the full EEG frequency spectrum (F1, C1, Pz, CPz, AFz, Fz, F2, FCz, CP2). The results proved that with each of the EEG signals is possible to identify emotional states in the participants using a smaller time window with game events and these events have a relation with the arousal-scores gave by the participants.

## 4. Discussion

Prior works have reported that higher frequency bands relate with emotions while using pictures, videos or recalling past experiences as emotional stimuli. For example, in [45], authors reported the presence of decrease of peaks in the alpha band for fear and sorrow emotions, in contrast of increase in peaks frequencies in the alpha band for joy and anger. Works using the SEED dataset [38], have reported that alpha, beta, and gamma had better emotion classification performance [46,47]. Their findings showed that for positive emotion, beta and gamma frequency bands energy increases, and for neutral and negative emotions beta and gamma frequency bands have lower energy. In addition, neutral emotions have higher energy on alpha band [48]. Other works support this findings using pictures as emotional stimuli [49], and using the DEAP dataset [50]. EEG traits correlated with arousal for the majority of the participants shows that emotions can be described from the theta band of the central electrodes (FCz, FC1, FC2, Cz, C1, C2, CPz, CP1, CP2), however, on an individual approach, when performing regression to predict emotional values with feature selection, the selected features for each participant showed that traits from the alpha, beta and gamma band were selected in higher rate than the theta band traits. In addition, some time domain features like complexity and standard deviation of the channel’s signals are selected in the majority of the participants.

Comparing with other works using videos as emotional stimuli [36,37,38,51,52], arousal and valence labels classification metrics (accuracy and F1 scores) have values around 0.5 and 0.8 using machine learning methods as support vector machines and k-nearest neighbors. Arousal label classifications scores have better results than valence label classifications. In our case, we used regression analysis to predict the arousal and valence scores. Our results showed error metrics higher than 0.4 but lower than 1.2 for MAE and the MSE showed values higher than 0.3 but lower than 3.5 for arousal scores, and error metrics higher than 0.7 but lower than 2.1 for MAE and the MSE showed values higher than 0.8 but lower than 4.0 for valence scores. As previous works, classification/prediction of valence labels/scores is more difficult than for arousal. This is not only reflected on the performance metrics but also in the EEG traits correlation with valence scores. This can be related to the process of rating overall positive and negative emotional experience on a task (video or videogame) that had diverse contents within the same long-time window. Depending on the content showed to the participant in a long time-window, the participant can have both positive and negative emotions on different moments inside the time window, making difficult to summarize the overall experience with one score and, at the same time, generalize the signal activation through the whole-time window, narrows the possibility of identify specific moments where each participant could feel different emotions related to high or low valence responses. Although, the results are consistent with previous works, more studies that analyze emotional reactions and EEG traits under interactive virtual environments are needed to contrast our findings using the same emotional stimuli nature.

Some ERP components elicited by visual stimuli can be modulated when the images have emotional content: P1 modulated by the presentation of emotional faces, N1 modulated by both pleasant and unpleasant stimuli component may represent early processes in the evaluation of emotional stimuli, N170 and P2 modulated by emotional faces [25]. However, when playing video games, several brain areas are stimulated (occipital lobe for visual processing, parietal and temporal lobes from auditory stimuli, and the frontal lobe for emotional processing, concentration and decision making) [17], and is important to study how particular events can be represented by EEG activity, some works have focus their analysis on the relation of EEG and events [53,54]. When we consider EEG activation in presence of game events, we found strong correlation of traits related to theta, alpha and beta frequency bands across all participants, with the strongest scores belonging to the theta and the alpha bands from electrodes positioned on the front, central and occipital brain regions. Our findings are consistent with works that also analyze game events and the related EEG activation. In [12], the authors collected information about a variety of relevant game events, presumably negative events had strong responses with delta band signal component and delta/theta power increase, together with a strongly differentiated ERPs. In addition, rewarding events caused an increase in low delta signal component and high delta power with a robust ERP peaking and plateauing around the time of the P2 component. In our experiment, we found P2 characteristics on the EEG signals in presence of positive events, where before the event and after 0.5 s of the event the signal stabilizes. In contrast, we found a pronounce peak in amplitude in presence of negative events, and a decrease in amplitude (negative amplitude) before the occurrence of the event, this suggest that the participants can predict when a negative event is about to occur (0.5 s before the event).

Although, the number of participants in our study is relatively small, the patterns found on the EEG signals across subjects suggest a common activation and perception for both positive and negative events, what is worth to highlight is that emotion appraisal (arousal and valence responses) is an individual process that depends on past experience, memories and cognitive process related to each participant, this can explain why not too many common EEG traits corralled among participants, but still there was higher number of EEG traits that correlated in an individual level, for this kind of scenarios, participant tailored approaches to identify emotional reactions are more suitable due to the consideration of individual characteristics and reactions [7,8]. It is necessary to conduct more experiments with a higher number of participants where characteristics as age, culture, sex, etc., can be considered to analyze the influence on emotion recognition process.

It is important to study how the events are also related with other cognitive process that can also influence emotion under HCI scenarios, in this work, we only correlated the EEG information with emotional data (self-assessment answers or game event nature), but it is worth to explore other cognitive process that complement the emotion recognition process. In addition, it is worth to explore the EEG characteristics in other HCI scenarios where emotional information can be measure and analyze, not only in the videogame field, where time information related to task/events can be analyzed together with the appraisal of the emotional content. Our next objective is to implement different classification/regression algorithms to improve the recognition online pipeline and find more robust result while targeting arousal–valence scores including game events’ information to improve the process due to the good results obtained.

## 5. Conclusions

We found that it is possible to identify emotional reactions using EEG traits using videogames as an emotional stimulus. Videogames are powerful tools to elicit emotions due to the combination of digital media as music, picture, videos, game mechanics and story-telling, with the interaction with virtual environments, becoming an efficient tool to study emotional reactions under HCI scenarios. It is important to analyze how to identify emotional reactions under interactive scenarios using EEG data that allows to detect patterns or correlated information with answers and reactions related to emotion.

The emotional reactions came represented in self-assessment responses, and also, in game time events. For self-assessment responses, theta, alpha, beta and gamma bands of electrodes from the central, occipital, and temporal regions allows to predict arousal values better, in contrast, with the performance of predicting valence values. Addressing game events, we found that EEG traits related with the theta, alpha and beta band had strong correlations. Also, distinctive event-related potentials where identified in the presence of both types of game events that correlated with the emotional responses.

## Figures and Tables

**Table 1:** Signal traits calculated for the physiological signals.

Article	EEG Characteristics	Video Game and Measured Emotions	Game Play Time Window	Emotional Reference Information	Game Time Event Analysis	Participants
[14]	14 channels	Train Sim World—boring, Unravel—calm, Slender—The Arrival—horror, and Goat Simulator—funny.	5 min	Arousal/Valence Self-Assessment Manikins (SAM)	No	28
[15]	9 channels	Four architectural environments designed based on Kazuyo Sejima’s “Villa in the Forest” modifying illumination, color, and geometry. High and low arousal and valence states.	1.5 min	Arousal/Valence Self-Assessment Manikins (SAM)	No	38
[16]	24 channels	Candy Crush and Stickman Archers. Happiness, sadness, surprise, anger, disgust and neutral	10 min	Visual inspection of facial expressions	No	35
[17]	19 channels	Tetris: medium condition, easy condition, hard condition	5 min	Arousal/Valence Self-Assessment Manikins (SAM)	No	14

**Table 2:** Signal traits calculated for the physiological signals.

Type of Feature	Feature Name
Time domain features	Picard parameters [31,32]: mean, standard deviations of the physiological signal, max/min ratio of the EEG signals. Higher order statistics [33]: skewness measures the degree of asymmetry of a distribution around the signal’s mean. Kurtosis is the measure of relative heaviness of the tail of a distribution with respect to the normal distribution. Hjorth variables [34,35]: activity represents the signal power by the variance of a time function. Mobility represents the mean frequency or the proportion of standard deviation of the power spectrum. Complexity represents the change in frequency comparing the signal’s similarity to a pure sine wave, the value converges to 1 if the signals are similar.
Frequency domain features	Power spectral density (PDS) [31,36,37,38] by Welch’s method (time window = 512 samples corresponding to 1 s, on the theta (4–8 Hz), alpha (8–12 Hz), beta (12–30 Hz) and gamma (30–47 Hz) bands for each electrode) [36]. PS-ASM between the 27 pairs of electrodes in the five bands were calculated [36]. Differential entropy (DE) equivalent to the logarithm of the energy spectrum [39,40]. DE can be defined as the entropy of continuous random variables and is used to measure its complexity, and is equivalent to the logarithm of the energy spectrum in a certain frequency band for a fixed length EEG sequence ([41]). DASM and RASM were calculated as the differences and ratios between the DE of the 27 pairs of asymmetry electrodes [36].

**Table 3:** Arousal and valence means and standard deviation for the 24 game levels.

	Game Level	Arousal		Valence	
		Mean	Std	Mean	Std
HAHV	N–01	7.05	0.45	7.23	1.24
	N–02	7.29	1.03	6.65	1.30
	N–03	6.92	0.99	6.93	1.19
	SU–01	8.01	0.68	6.71	1.64
	SU–02	7.99	1.22	6.40	1.60
	SU–03	7.24	0.75	5.74	1.59
HALV	H–01	7.84	0.89	4.62	2.52
	H–02	6.64	1.06	3.92	1.94
	H–03	6.55	1.94	4.71	2.56
	OA–01	7.64	1.13	4.56	2.33
	OA–02	6.81	1.97	4.62	2.31
	OA - 03	6.63	1.34	4.34	2.10
LAHV	E–01	4.02	1.78	6.80	1.23
	E–02	4.79	1.86	7.23	1.75
	E–03	4.51	1.76	6.71	1.45
	WS–01	4.18	1.71	5.27	1.73
	WS–02	3.42	1.12	5.44	1.24
	WS - 03	3.22	1.48	5.37	1.25
LALV	SD–01	3.50	1.85	5.15	1.59
	SD–02	3.60	1.46	5.03	1.32
	SD–03	2.59	1.49	4.69	1.51
	WT–01	2.17	0.96	4.37	2.26
	WT–02	1.94	1.22	5.27	1.35
	WT–03	2.18	1.64	4.94	2.08
--	Final Mission	7.05	0.45	7.23	1.24

**Table 4:** Number of electroencephalography (EEG) traits correlated with arousal and valence score: (**a**) for each participant, (**b**) traits common among participants.

a Individual Traits Correlated for Each Participant				b Number of Traits Correlated Common among Participants		
Participant	Gender	Arousal	Valence	Number of Participants	Arousal	Valence
		Num. of Traits	Num. of Traits		Num. of Traits	Num. of Traits
1	Male	223	200	1/10	461	480
2	Male	245	9	2/10	260	82
3	Male	207	10	3/10	155	0
4	Female	265	8	4/10	79	1
5	Male	287	323	5/10	35	0
6	Male	272	16	6/10	10	0
9	Male	146	13	7/10	4	0
10	Female	254	60	8/10	9	0
11	Male	140	7	9/10	2	0
12	Female	106	2	10/10	3	0
Total		2145	648	Total	2145	648

**Table 5:** Number of positive and negative events per participants.

Events	Participants									
	1	2	3	4	5	6	7	8	9	10
Positive	470	428	437	409	465	478	467	445	450	320
Negative	85	121	94	130	114	91	117	114	151	171

**Table 6:** Classification performance scores of positive and negative events per participants.

			Events							
			Training						Test	
Num. of Participants	Gender	N. Traits	Acc		F1		AUC		Acc	F1
			Mean	Std	Mean	Std	Mean	Std		
1	Male	21	0.97	0.05	0.98	0.03	0.99	0.01	0.91	0.94
2	Male	2 (Pz)	0.99	0.03	0.99	0.02	0.99	0.0	0.99	1.00
3	Male	2 (PO3)	0.99	0.04	0.99	0.04	0.99	0.01	0.97	0.98
4	Female	2 (Pz)	0.99	0.02	1.00	0.01	0.99	0.0	1.0	1.0
5	Male	2 (Oz)	0.99	0.03	0.99	0.02	0.99	0.0	0.98	0.98
6	Male	2 (POz)	0.99	0.02	0.99	0.01	0.99	0.01	0.98	0.99
9	Male	2 (Pz)	1.0	0.01	1.0	0.01	1.0	1.0	1.0	1.0
10	Female	2 (P8)	1.0	0.01	1.0	0.01	0.99	0.0	1.0	1.0
11	Male	2 (PO4)	0.99	0.02	1.0	0.02	0.99	0.0	0.98	0.98
12	Female	2 (P3)	0.99	0.05	0.99	0.05	0.99	0.0	1.0	1.0

**Figure A4:**
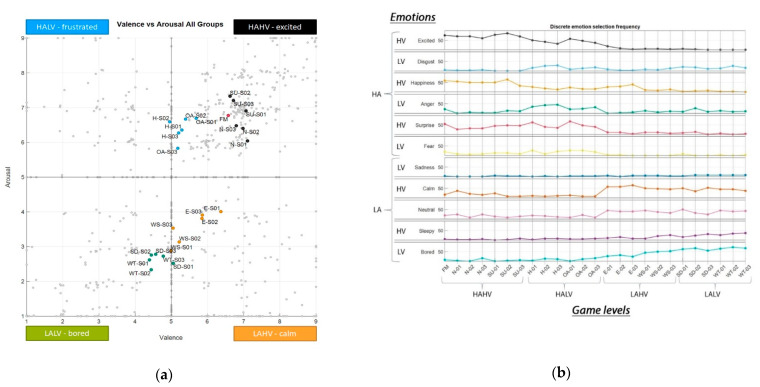
(**a**) Arousal–valence dispersion. Levels names: N—normal, SU—speed up, H—hard, OA—only asteroids, E—easy, WS—without speed, SD—speed down, WT—without tokens. Stage names: S01—Stage 01, S02—Stage 02, S03—Stage 03. The colors are related to the emotional quadrants that we intent to induce: HAHV—black, HALV—blue, LAHV—orange, LALV—green. (**b**).

## Data Availability

The data presented in this study are available on request from the corresponding author.

## Appendix A

In this section, we explain details about the videogame design to elicit emotions and the result of the test phase with two control groups.

### Appendix A.1. Selection of Target Emotions

With this videogame, we wanted to target different emotions to study the participant’s emotional reactions in HCI scenarios. We selected four emotional states from the Russell’s circumflex model of emotion [18] (Figure A1a), where emotions are defined by two scores distributed in a two-dimensional plane: valence (the positive and negative degree of the emotion represented by the *x* axis) and arousal (intensity of the emotion represented by the y-axis represents).

### Appendix A.2. Videogame Frameworks

The game design was based in two videogame development frameworks (the Mechanics-Dynamics-Aesthetics (MDA) framework, and the 6–11 framework), and the concept of flow in games. MDA framework [55], breaks the games into their distinct components (rules, system and fun) and its design counterparts (mechanics, dynamics and aesthetics), where aesthetics become the main element to create emotions in the participants using the game mechanics and dynamics. The 6–11 framework [56] defines six basic emotions and eleven instincts that interact with each other to elicit “fun” using game events that affects and influence the player’s experience. Finally, flow [57] refers to the player’s ability to choose and control actions inside a videogame environment. To be on flow, the game must achieve a balance between challenges and players’ abilities: if the game is too challenging and the player’s abilities are not that high, the game experience becomes anxious, in contrast, if the game is not that challenging and the player’s abilities are too high, the experience becomes boring (Figure A1b).

**Table A1:** Level characteristics (* compared to normal level).

Level	Emotions	Ships Characteristics	Tokens Characteristics	Tokens Stage 1	Tokens Stage 2	Tokens Stage 3
Normal	HAHL Excitement	Normal Speed Normal Controls Accelerate Option	Normal Speed	Astronauts Asteroids	Astronauts Asteroids Special Asteroid	Astronauts Asteroids Enemies
Speed Up		Normal Speed Normal Controls Accelerate Option	Speed Gradually Increases	Astronauts Asteroids	Astronauts Asteroids Special Asteroid	Astronauts Asteroids Enemies
Hard	HALV Frustration	Speed Gradually Decreases Inverted Controls every 10 s Deaccelerate Option	Normal Speed Larger Size of Asteroids Smaller Astronaut’s Collision Region If Collision, The Size of the Negative Tokens Increases	Astronauts Big Asteroids Asteroids	Astronauts Big Asteroids Special Asteroid	Astronauts Big Asteroids Enemies
Only Asteroids		Normal Speed Normal Controls FOV Gradually Decreases Accelerate Option	Speed Gradually Increases Respawn Time Gradually Decreases No Good Tokens Inclusion of Larger Sized Asteroid If Collision, the Size of FOV Decreases	Big Asteroids Asteroids	Big Asteroids Asteroids Special Asteroid	Big Asteroids Asteroids Enemies
Easy	LAHV Calm	Normal Speed Normal Controls Accelerate Option	Decrease Speed * No Negative Tokens	Astronauts Small Coins Big Coins	Astronauts Small Coins Big Coins	Astronauts Small Coins Big Coins
Without Speed		Normal Speed Normal Controls No accelerate Option	Decrease Speed *	Astronauts Asteroids	Astronauts Asteroids Special Asteroid	Astronauts Asteroids Enemies
Speed Down	LALV Bored	Higher Decrease Speed Normal Controls Accelerate Option	Higher Decrease Speed * Spawn from the Middle of the Screen	Astronauts Asteroids	Astronauts Asteroids Special Asteroid	Astronauts Asteroids Enemies
Without Tokens		Higher Decrease Speed Normal Controls Accelerate Option	None	None	None	None
Final Mission	–	Speed Depends on Power Ups Normal Movement Controls Shooting Option	Normal speed Different trajectories Respawn in pair	Asteroids Special Asteroids Enemy type 01 Enemy type 02		

### Appendix A.3. Final Mission

The final mission has two outcomes: win or lose. When the final boss’s life bar reaches cero, the game will be over and the player will win the game; in contracts, when the player’s life bar reaches zero, the game will be over and the player will lose, finishing the session in both cases. To complete the final mission, the speed control is changed to a shooting rate control, and the players will have a series of power ups and upgrades (represented as number of guns, type of ammunition, and number of shields), related to their previous stages’ performance. The final boss’s movement is limited to the *x* axis scroll movement from the screen right side to left side. To create tension while the final boss is approaching the player’s position, the speed will be lower than the regular tokens speed on the main stages’ levels. Beside the final boss, there are 4 types of enemies that appear in pairs on a random *y* position in the right part of the screen, have a scrolling movement from right to left, and have a distinctive trajectory (Figure A3).

### Appendix A.4. Videogame Evaluation

Two participant groups tested the videogame, and emotional questionnaire answers were analyzed. We implemented a first version of the videogame, gathering the emotional questionnaires’ results from a control group of participants [58]. After obtaining and analyzing the result, we designed and implemented a second version of the videogame and analyzed the emotional questionnaires’ results from a participants group from the control group that tested the first version of the videogame. On this opportunity, 17 students (females—4, males—13), ages ranging between 21 and 25 (mean 22.70 ± 1.31) tested the game. After the first trial, we tested the same version of the videogame with a second group of participants, to analyze the results when the participant is not familiar with the content and is watching the game for the first time, 13 students (females—2, males—11), ages ranging between 20 and 25 (mean 22.0 ± 1.78).

Both participant groups performed one session of gameplay in the computational laboratories from the Pedagogical and Technological University of Colombia in a simultaneous scheme using personal computers and earphones. At the end of the sessions, the researchers collected a log file from each of the participant’s gameplay, containing emotional questionnaires’ answers, behavioral and performance data. All participants agreed to participate in the experiment by signing an informed consent.

### Appendix A.5. Arousal–Valence Dispersion

We analyzed the arousal and valence dispersion among the three groups. In Figure A4a, the arousal and valence scores from all the participants are represented in a two-dimensional plane. According to the arousal–valence dispersion, the participant reported different emotional states within each level, the obtained means are related to a generalized emotional experience among the participants, and they work as a reference on how each level is eliciting emotional states. The dispersion shows that it is possible to have a wide range of arousal–valence scores among the participants from the 2 groups, and each of the designed levels can induce the fourth emotional targets selected from the game design.

For normal and speed up levels (N, SU, black dots), HAHV scores were reported throughout the three stages. It is possible to see that the mean values are similar and the majority of the distribution is located in the HAHV quadrant, leading to infer that the excited emotion was achieved over the six levels. For hard and only asteroids levels (H, OA, blue dots), the arousal scores aimed to represent HA responses. However, it is possible to see that valence scores distribution is located between HV and LV quadrants, making the reference means to be located close to the neutral valance value, which lead to infer that the six levels produced two kind of emotions, excited and frustrated.

For easy and without speed levels (E, WS, orange dots), LA responses were reported by the participants. For easy levels, HV scores were higher than the without speed levels’ valence scores. Without speed levels’ mean reference values are located close to the neutral valence value. In general, the easy levels’ mean reference values showed that calm emotion was induced by the six levels, and the without speed levels’ mean reference values showed that bored and calm emotion were induced in the participants. For speed down and the without tokens levels, LA responses were reported by the participants. Valence responses distribution is located between the two valence quadrants making the mean reference values to be located in the LV quadrant but close to the neutral valence value. In general, bored emotion was achieved over the six levels. Finally, for the final mission level, most of the answer distribution was located in the HAHV quadrant, leading to infer that excitement is the emotion felt by the participants while playing this last level.

### Appendix A.6. Discrete Emotion Selection

In Figure A4b, the discrete emotion selection percentage for each level is shown, the percentage correspond to the total of participants from the 3 groups. The figure shows that for the game levels related with HA emotions (normal, speed up, final mission), the selection of emotions as excited, and happiness were higher than 50% of the participants. Anger, discuss, surprise and fear had an increase selection between the game levels related with HALV (hard, only asteroids). For game levels related with LA (easy, without speed, speed down, without tokens), the selection of emotions as neutral, calm, and bore were higher than or close to 50% of the participants. Bored and sleepy emotions got a selection increase between the levels related with LALV (SD, WT). The graphs behavior show that it is possible to have a wide range of discrete emotions among the participants from the 3 groups, and each of the designed levels can induce emotions related to the arousal—valence quadrants from the game design. For all the levels, fear, disgust and sadness emotions were selected in some cases, but the selection does not represent more than third percentage of the participants.

For HAHV game levels in overall, high valence emotions were reported for normal levels. For speed up levels, high arousal and high valence emotions were reported. For HALV game levels in general, the emotions selected by the participants in greater degree were excited and surprise, followed by anger. The results showed that it is possible to obtain frustrated related emotions with these levels as anger. For LAHV game levels in overall, low arousal emotions were chosen in these levels. We see that the majority of the participants choose calm, and in less degree neutral, as felt emotions across the three stages for Easy level. In contrast, for without speed level the selection was divided between calm, bored and neutral emotions depending on the stage. For LALV game levels in overall, low arousal emotions were chosen. We see that the majority of the participants choose bored as a felt emotion across the three stages for both levels, followed by calm, neutral and lastly sleepy emotion selection. Finally, for the final mission level, participant reported have felt excited (81.57%), happiness (60.52%) and surprise (57.89%) emotions.

### Appendix A.7. Repeated-Measures ANOVA between Level Stages of Each Group

We performed a repeated analysis of variance (RANOVA), followed by a Tukey H SD (HSD—honestly significantly different) post hoc test to identify the stages that had a significant difference (*p* < 0.05) within each group. After identifying the stages, we performed a two tailed t- test between the variables of the stages that had significant differences, to identify which emotional questionnaires responses had a significant difference between the same levels on different stages.

For Group 1, in only asteroids levels, the $h_{0}$ was rejected for neutral between Stage 2 and Stage 3, a higher percent of participants selected neutral emotion as a felt emotion for Stage 2 (23.53%) in contrast with Stage 3 (0%).

For Group 2, in Speed up levels, the $h_{0}$ was rejected for fear emotion between Stage 1 and Stage 3, a higher percent of participants selected fear as a felt emotion for Stage 3 (30.77%) in contrast with Stage 1 (0%).
